# Characterization of Thermoplastic Starch Plasticized with Ternary Urea-Polyols Deep Eutectic Solvent with Two Selected Fillers: Microcrystalline Cellulose and Montmorillonite

**DOI:** 10.3390/polym15040972

**Published:** 2023-02-16

**Authors:** Magdalena Zdanowicz, Kamila Sałasińska

**Affiliations:** 1 Center of Bioimmobilisation and Innovative Packaging Materials, Faculty of Food Sciences and Fisheries, West Pomeranian University of Technology, Szczecin, Janickiego 35, 71-270 Szczecin, Poland; 2 Faculty of Materials Science and Engineering, Warsaw University of Technology, Wołoska 141, 02-507 Warsaw, Poland; 3 Central Institute for Labour Protection—National Research Institute, Department of Chemical, Biological and Aerosol Hazards, Czerniakowska 16, 00-701 Warsaw, Poland

**Keywords:** cone calorimetry, deep eutectic solvents, fillers, fire behavior, microcrystalline cellulose, montmorillonite, thermoplastic starch

## Abstract

The aim of the study was to prepare and characterize composite materials based on thermoplastic starch (TPS)/deep eutectic solvent (DES). Potato starch was plasticized with ternary DES: urea:glycerol:sorbitol and modified with the selected fillers: microcrystalline cellulose and sodium montmorillonite. Films were prepared via twin-screw extrusion and thermocompression of the extrudates. Then, the physicochemical properties of the TPS films were examined. The ternary DES effectively plasticized the polysaccharide leading to a highly amorphous structure of the TPS (confirmed via mechanical tests, DMTA and XRD analyses). An investigation of the behavior in water (swelling and dissolution degree) and water vapor transmission rate of the films was determined. The introduction of the two types of fillers resulted in higher tensile strength and better barrier properties of the composite TPS films. However, montmorillonite addition exhibited a higher impact than microcrystalline cellulose. Moreover, a cone calorimetry analysis of the TPS materials revealed that they showed better fire-retardant properties than TPS plasticized with a conventional plasticizer (glycerol).

## 1. Introduction

Starch plasticizers are low molecular polar compounds that enable the polysaccharide processing into more amorphous “plastic-like” materials called thermoplastic starch (TPS). The TPS materials can be obtained e.g., via gelatinization of starch in aqueous media casting and evaporation of water or via extrusion [1]. The modification via extrusion is solventless (water, more often in form as starch moisture, acts as a co-plasticizer), does not require water evaporation from casted materials and allows processing the material on a large scale in a short time, which is crucial issue from the industrial point of view. Due to native starch having a glass transition close to its degradation temperature, it needs a special additive to facilitate its modification. Starch plasticizers disrupt H-bonding between polysaccharide chains and form new ones with OH groups of anhydroglucosidic units of the polymer. The most common starch plasticizers are glycerol (G) or other higher polyalcohols (sorbitol, maltitol) [2,3,4], amides [5,6,7], sugars [8,9] or carboxylic acids (in a smaller amount, they can also act as crosslinkers) [10]. In the last two decades, interest in ionic liquids (ILs) as polysaccharide processing media rapidly increased [11,12,13]. Due to the high cost of ILs and often non-green character, deep eutectic solvents are used as more eco-friendly tailorable, alternative media not only for starch treatment (e.g., plasticizing, dissolution) [14,15,16,17,18,19] but also for polysaccharides processing such as their extraction [20,21], biomass pretreatment (e.g., delignification, fractionization, and separation) [22,23,24]. DES are mixtures that exhibited phase transition temperatures at much lower temperature that their components. In comparison with ILs, DES are easy-to-prepare media from “greener” (often from natural sources) and cheaper components obtained via mixing [15].

TPS materials are highly hydrophilic, thus some fillers are introduced into polymer matrix not only to increase barrier properties and decrease moisture sensitivity but also to facilitate mechanical properties or add some functionality (i.e., conductivity or antimicrobial properties [25]). However, there are only few works that describe studies of mutual interactions between starch, DES as a plasticizer and a filler [26,27,28,29,30].

In the previous work, we confirmed that DES based on urea, glycerol, and sorbitol (UGS) effectively plasticizes starch via thermocompression [31]. In this work, ternary DES UGS is used for starch processing via extrusion for the first time. Moreover, two selected fillers were added: mineral sodium montmorillonite (M) and organic microcrystalline cellulose (C) to investigate their influence on TPS/DES materials. Fillers were added in two concentrations: 5 and 10 pph on dry starch. Mechanical tests, sorption tests, water vapor transmission rate (WVTR) determination, and dynamical mechanical thermal analysis (DMTA), and X-ray diffractometry (XRD) for TPS and TPS composites were performed. In our previous work we studied the fire behavior of TPS plasticized with ternary DES based on choline chloride:resorcinol:urea with lignin addition [30]. However, there are only few works describing the fire behavior of TPS materials [32,33,34]. Due to M being known as a fire retardant, a cone calorimetry analysis for TPS with clay addition was performed. The results were compared to starch plasticized with a conventional plasticizer (glycerol).

## 2. Materials and Methods

### 2.1. Materials

Potato starch with a moisture content of 15.5 wt% (average molecular weight 4.37 × 107 g/mol; 29.7 wt% amylose content) was supplied by Nowamyl SA (Nowogard, Poland). Urea—U (≥98%) and glycerol—G (99%) were purchased from Chempur (Poland) and sorbitol—S (99%) from Alfa Aesar (Kandel, Germany). As fillers were used one selected organic filler—microcrystalline cellulose—C (particle size 20 µm purchased from Sigma–Aldrich), and sodium montmorillonite—M (Cloisite Na^+^, Southern Clay Products, Gonzales, TX, USA) as an inorganic filler.

### 2.2. Eutectic Mixtures Preparation

Eutectic mixtures were prepared as follows: selected components: U, G and S at molar ratio 2:1:1, respectively, were placed in a glass reactor heated up to 95 °C and stirred until the homogenous pellucid liquid was obtained; then the mixture was poured into a glass vial and placed in a vacuum chamber (105 °C, 250 mbars, 1 h) to remove the remaining moisture and then kept in sealed vials. The melting point of the DES UGS according to DSC analysis is 59 °C [31].

### 2.3. Preparation of TPS Films via Extrusion

Preparation of TPS films was as follows: premixtures of starch, filler and DES were processed after 1 day of storage. The systems were extruded with a laboratory twin-screw co-rotational extruder with an L/D ratio of 40:1 and a screw diameter of 16 mm (PRISM Eurolab Digital, Thermo Electron Co., Waltham, MA, USA). The temperature profile from the feed throat to the nozzle was 80/100/110/120 × 7 °C, and the screw speed was maintained at 150 rpm. After processing, extrudates were pelletized with a granulator (Prism Varicut), obtaining ca. 3 mm pellets. After 14 days of storage at ambient conditions in sealed PE bags, pellets were thermocompressed at 120 °C under 12 tons (153 bars), cooled down under pressure to ca. 85 °C and stored in a climate chamber (25 °C, RH 50%) for 48 h before testing.

### 2.4. Mechanical Tests

Mechanical tests for TPS films were performed using Instron 5982 (Instron, Norwood, MA, USA, load cell 1 kN) according to ASTM D822-02 standard. The films (thickness 0.50–0.65 mm) were cut into 10 mm wide, 120 mm long stripes. The initial grip separation was 50 mm, and the crosshead speed was set to 10 mm/min. At least eight replicated samples for each system were tested and the mechanical parameters (EB—elongation at break, TS—tensile strength and YM—Young’s modulus) were calculated with the Bluehill 3 software.

### 2.5. DMTA Measurements

The measurements were carried out with a film tension clamp at a frequency of 1 Hz, a heating rate of 3 °C/min, and a temperature range from −90 to 140 °C using Dynamic Mechanical Analyzer Q800 (TA Instruments, New Castle, DE, USA). The analysis was conducted twice for each material.

### 2.6. Microstructure Analysis

The microstructure of composites was examined using a scanning electron microscope TM3000 (Hitachi, Tokyo, Japan), equipped with a backscattered electron detector (BSE). The samples were fixed to carbon tape and coated with gold using POLARON SC7640 (Quorum Technologies Ltd., Newhaven, UK). Images were taken with an accelerating voltage of 5–15 kV. The magnification of 1000× was used.

### 2.7. X-ray Diffraction Analysis of TPS Films

The crystallinity of TPS films was analyzed using the XRD technique (X’pert Pro, PANalytical, Almelo, The Netherland, operated at the CuK(alfa) wavelength 1.54 Å). The d-spacing was calculated from the Braggs formula (2 d sinθ = n λ, where λ = 0.154 nm) being the order of reflection, θ the angle of refraction).

### 2.8. Moisture Sorption, Water Sorption and Swelling Degrees

Samples (three samples for each test) with dimensions of 20 mm × 20 mm were prepared for moisture sorption and swelling tests. The test was performed as in previous work [35]. Samples were placed in a vacuum dryer (250 mbars) at 65 °C/24 h. Mass of dried samples, as well as stored in a climate chamber and kept at 25 °C and 75% RH (for moisture sorption evaluation) or immersed in distilled water for 24 h (for swelling behavior investigation), were determined.

### 2.9. Forced Flaming Fire Behavior

Burning behavior was evaluated by cone calorimeter examinations conducted on Fire Testing Technology apparatus (East Grinstead, UK,) following the ISO 5660-1 and ISO 5660-2 procedures. The horizontally oriented samples were irradiated at a heat flux of 35 kW/m^2^, and spark ignition was employed to ignite the pyrolysis products. An optical system with a silicon photodiode and a helium-neon laser provided a continuous survey of smoke. The burning process during tests was photographed using a digital camera, EOS 400 D, from Canon Inc. (Tokyo, Japan).

### 2.10. Statistical Analysis

Statistical analysis of the mechanical test data for extruded samples was subjected to one-way analysis of variance, and the significant difference was determined by the significance difference test (*t*-Student’s test).

## 3. Results

### 3.1. Mechanical Test Results

Table 1 lists mechanical tensile test results for extruded TPS films. It can be seen from the collected data that nonionic ternary DES based on urea, glycerol, and sorbitol led to better mechanical properties than choline chloride/U DES [27] or starch plasticized only with glycerol (TPS-G) [36]. The introduction of S into plasticizing mixtures can increase the tensile strength of the TPS films due to a higher amount of H-bonds formation. TS value is similar to TPS/DES obtained via only thermocompression (5.3 MPa). However, EB is about 50% higher [31]. It can be caused by better distribution of plasticizer in the polymer matrix and more untangled polymer chains formation during extrusion. Comparing TPS film with the composites, both additives affected mechanical properties leading to increased TS and YM and decreased EB. However, adding 5 pph of the filler did not affect the TS significantly (*p* < 0.05). The higher content of the filler caused a higher TS value. In turn, 10 pph of M addition led to slightly higher TS than 10 pph of C.

Based on the literature results for TPS composites, the increase in tensile strength depends on the plasticizer as well as the type of filler. For example, for TPS composites plasticized with glycerol where unmodified M was added, an increase in TS was from 6.0 to 7.6 MPa for 10% of M addition [37] or from 6.9 to 7.3 MPa for 8% addition of M [38] but when M is activated with plasticizer the increase in TS is more pronounced, due to intercalation of M platelets with the modifier [7,39,40]. Interestingly, adding the filler to the TPS matrix at high content (5 and 10 pph) did not affect it. A more discernible influence of the additive on TPS/DES was registered for our previous work, where TPS/ternary DES (choline chloride:resorcinol:urea) was prepared with dissolved lignin presence [30]. It could be caused by better distribution of lignin that was in dissolved form.

### 3.2. Dynamical Mechanical Thermal Analysis (DMTA) Results

**Figure 1:**
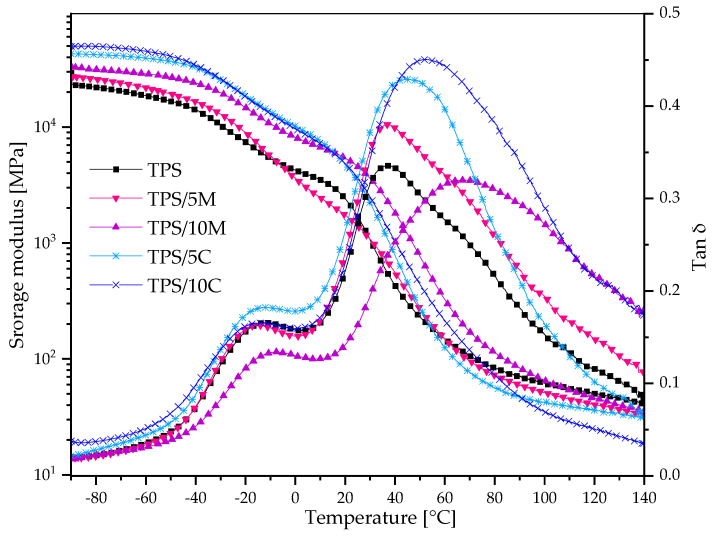
Storage modulus and Tan δ curves of TPS films.

In this work, DMTA is represented with tan delta and storage modulus curves presented in Figure 1. The TPS films revealed two main relaxation regions. The first with maxima at a range from −18 to −12 °C is related to secondary β-relaxation (T_β_) of the domain rich in plasticizer’s molecules, and the second α-relaxation (T_α_) with higher intensity (maxima at range 36–63 °C) and is assigned to a polysaccharide-rich domain. Similar results were also observed for TPS/UGS obtained via thermocompression [31]. The β-relaxation for starch plasticized with ternary DES occurred at a much higher temperature than glycerol (−52 °C) and even quite higher than DES based on sorbitol and choline chloride (−21 °C) [41], indicating that sorbitol with urea and glycerol formed stronger H-bonding than with choline chloride. It can result from U presence due to the amine group can form stronger hydrogen bonds than choline salt. Films with the fillers exhibited much higher temperatures of α-relaxations. It is caused by a more rigid structure with restricted mobility of polymer chains with the fillers [2].

In the case of E’ curves, there is a rapid drop of the parameter at ca. 20 °C for TPS without additive and at a range of 20–28 °C depending on the filler’s type and content. This E’ drop is assigned to a region of glass transition of the TPS. In the case of composite films E’ curve at higher temperatures is under TPS without filler. This behavior can be caused by clay’s platelets that slip between polymer/plasticizer matrix [27]. Moreover, applied plasticizer as a eutectic mixture may facilitate this behavior. Low E’ values of composite films at higher temperatures indicated that filler presence does not affect the processing of TPS/DES. The impact of the solid fillers presented in this work is more noticeable than for TPS/DES with lignin addition where the additive was introduced into the polymer matrix in dissolved form in the DES [30].

### 3.3. Behavior of TPS Films in Moisture, Water, and Determination WVTR

The swelling degrees for extruded TPS films are in the range of 71.1–87.4, and these values are lower than TPS/UGS obtained via thermocompression (221%) (Table 2) [31]. Introducing the fillers into the polysaccharide matrix lowered swelling and dissolution in water, moisture sorption as well as WVTR values. The higher the filler content, the lower values of the parameters. Comparing the type of fillers, the aluminosilicate addition led to better barrier properties than microcrystalline cellulose.

### 3.4. SEM Results

**Figure 2:**
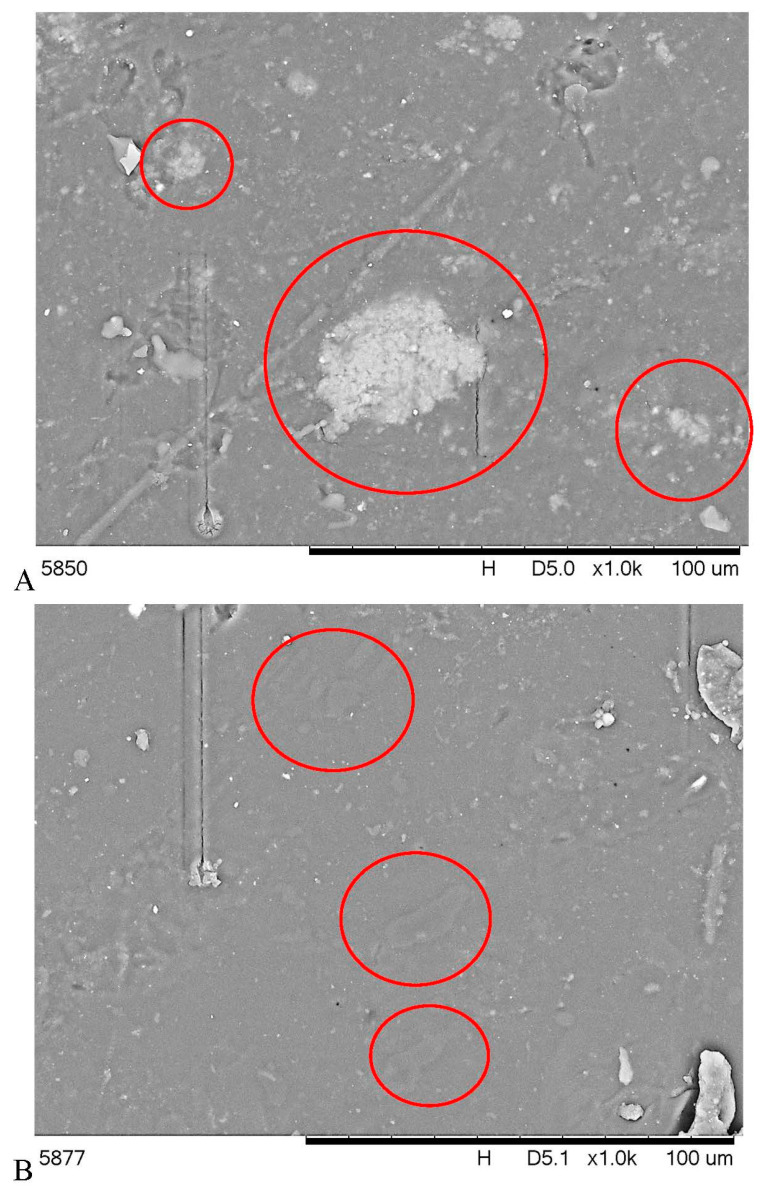
SEM micrographs for: (**A**)—TPS/10M and (**B**)—TPS/10C), filler particles are marked in red circles.

The morphology of the composites with the highest fillers amount is demonstrated in Figure 2. As can be seen, the material appearance is relatively smooth and homogenous, indicating a highly amorphous structure without swollen starch granules. Observation showed also relatively uniformly distributed particles; however, agglomerates, especially in the case of inorganic filler, were observed. Moreover, C and M particles significantly differed in size and shape. It can be noticed, especially for C, that the filler is well embedded in the matrix. In turn, a lack of pore at the interface between fillers and polysaccharide matrix, indicating good adhesion. This may be related to the improvement in the mechanical properties of composite materials.

### 3.5. X-ray Diffractometry Results

**Figure 3:**
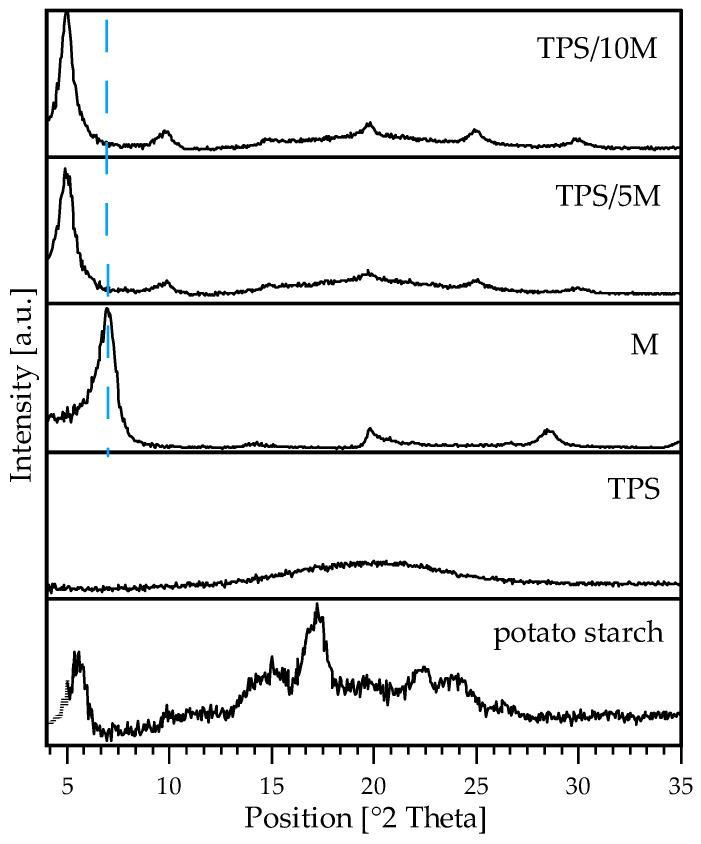
XRD patterns of native granular potato starch, TPS, sodium montmorillonite (M), and composite TPS films with 5 pph (TPS/5M) and 10 pph of M (TPS/10M).

Figure 3 shows diffractograms of native granular starch, The TPS film without filler, the pristine clay and the composite TPS films. The diffractogram of native potato starch granules reveals peaks at 5.4, 17.1, 19.5, 22.5, 24.0, and 26.0° that are characteristic of B-type semi-crystallinity of the polysaccharide. After extrusion with UGS, starch underwent plasticization effectively, which is confirmed with a flat diffractogram indicating the highly amorphous structure of TPS. For pristine M peak with high intensity at 7.0° is assigned to the interlayer spacing of clay platelets (d_001_ 1.26 nm). It can be seen that for the composite materials, there is a shift towards a lower contact value at 4.9° (1.80 nm) which indicates the intercalation of clay platelets in the polymeric matrix. This can result from better mechanical properties for TPS with M than with C (Table 1). The increase of the clay platelets’ distance can be caused by shearing forces during extrusion, facilitating the intercalation of processing starch with the presence of plasticizing agent causing swelling and partial untangling of polysaccharide chains between the filler platelets. For composite TPS, peaks appeared at 9.8, 14.9, 19.7, 24.8, and 30.1°, assigned to V-type crystallinity. The filler presence can induce the formation of post-processing crystallinity in small helical regions of starch.

### 3.6. Cone Calorimetry Results

**Figure 4:**
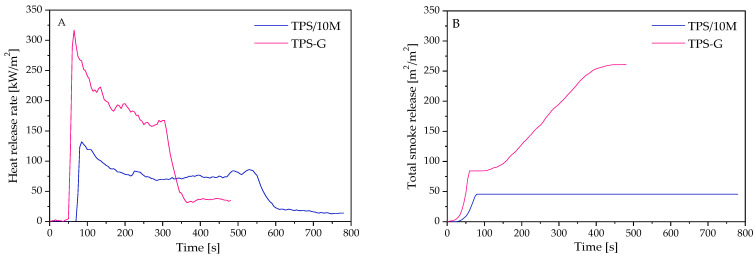
Representative curves of: (**A**)—heat release rate, (**B**)—total smoke release of investigated TPS materials.

Cone calorimetry is widely employed to assess the fire hazards of polymeric materials due to its capability to simulate real fire hazards [41]. According to the literature aluminosilicates are known as fillers with good fire retardant properties; therefore, the sample with montmorillonite addition (TPS/10M) was chosen for the investigation. Thermoplastic starch modified with a conventional plasticizer (TPS-G) was included in the test for comparison. The present study continues our work on the investigation of the fire retardant of thermoplastic starch with novel plasticizers based on DESs and its composites [30]. Figure 4 shows the heat release rate (HRR) and total smoke release (TSR) curves of TPS plasticized with ternary DES: UGS with 10 pph of sodium montmorillonite and TPS-G, while the detailed data are listed in Table 3.

During the cone calorimeter test, by oxygen consumption, the heat release rate (HRR) is specified as a crucial parameter to evaluate the intensity of fires [42]. As shown in Figure 4A, TPS-G exhibited a time to ignition (TTI) of 54 s, and a peak heat release rate (PHRR) of 317 kW/m^2^. The HRR curve presents a peak at the beginning of the burning and then gradually drops, which is characteristic of thick and able-to-char samples. The char-forming ability of thermoplastic starch, as one of the flame retardant mechanisms, was described in our previous work [30]. In turn, the curve of TPS/10M consists of two peaks at the beginning and end of burning, from which the first one yielded the PHRR. This curve course is typical for thick charring samples with additional peaks at the end of burning, and the second peak may be induced by cracking char or a growth in the effective pyrolysis [42]. Importantly, the PHRR of TPS/10M is much smaller compared to TPS-G and equal to 132 kW/m^2^, while TTI is as much as 79 s. Since the maximum average rate of heat emission (MARHE) is extrapolated from the maximum HRR, the value obtained for TPS/10M was also lower (reduction by 57%). The MARHE parameter is one of the essential indicators enabling flame spread evaluation. Another parameter used to assess the influence of modification on the fire growth rate is PHRR/t _PHRR_ [43], and the value obtained for the TPS/10M was three times lower compared to TPS-G. The delayed TTI, as well as reduced PHRR, MARHE and PHRR/t _PHRR_, demonstrate the improvement in the flame retardancy performance of TPS/10M.

Total heat release (THR) corresponds to the total heat output up to the defined point, which in this case, suits about 120 s after flaming burning has been completed. THR demonstrated a similar trend to PHRR, reaching 44 MJ/m^2^ and 60 MJ/m^2^ for TPS/10M and TPS-G, respectively. The lower THR observed for TPS/10M may follow from incomplete combustion caused by the formation of more char or reduced combustion efficiency [44]. Since the mass loss rate (MLR) and the effective heat of combustion (EHC) were also lower, both activities are assumable. Some charring polymers, which consist of carbon and heteroatoms, may reduce the EHC via accompanying fuel dilution [45]. The polymer containing a significant amount of carbon was a resource for the formation of char, while the sugar units degraded to non-flammable volatiles (H_2_O, CO_2_), favoring the development of its cellular structure. This effect was also supported by the presence of nitrogen from urea. The nitrogen compounds can promote the generation of a layer of gaseous products, which protects the material from the heat of the flaming zone, and volatile products can also act as radical interceptors [46]. Furthermore, nitrogen compounds form acids, which can catalyze the dehydration reaction of organic material, leading to char formation. The beneficial effect of montmorillonite on developing a durable and effective barrier preventing the exchange of mass and energy has been described in the literature [47,48]. Moreover, the influence of the lowered polymer share, as one of the main combustible components, in favor of an inorganic filler with higher thermal stability could not be excluded. Both carbonaceous char forming during the burning and inert residue from inorganic fillers reduce fuel release and, consequently, THR [42]. Compared to calcium montmorillonite, sodium montmorillonite used in the study is characterized by a greater ability to absorb water, which may be released during burning [49].

Excluding above mentioned key parameters, materials are assessed by the smoke generated, which can dramatically reduce the visibility during a fire, thus making it more difficult to escape. Smoke mainly consists of unstable carbon particles and cyclic compounds [41].

**Figure 5:**
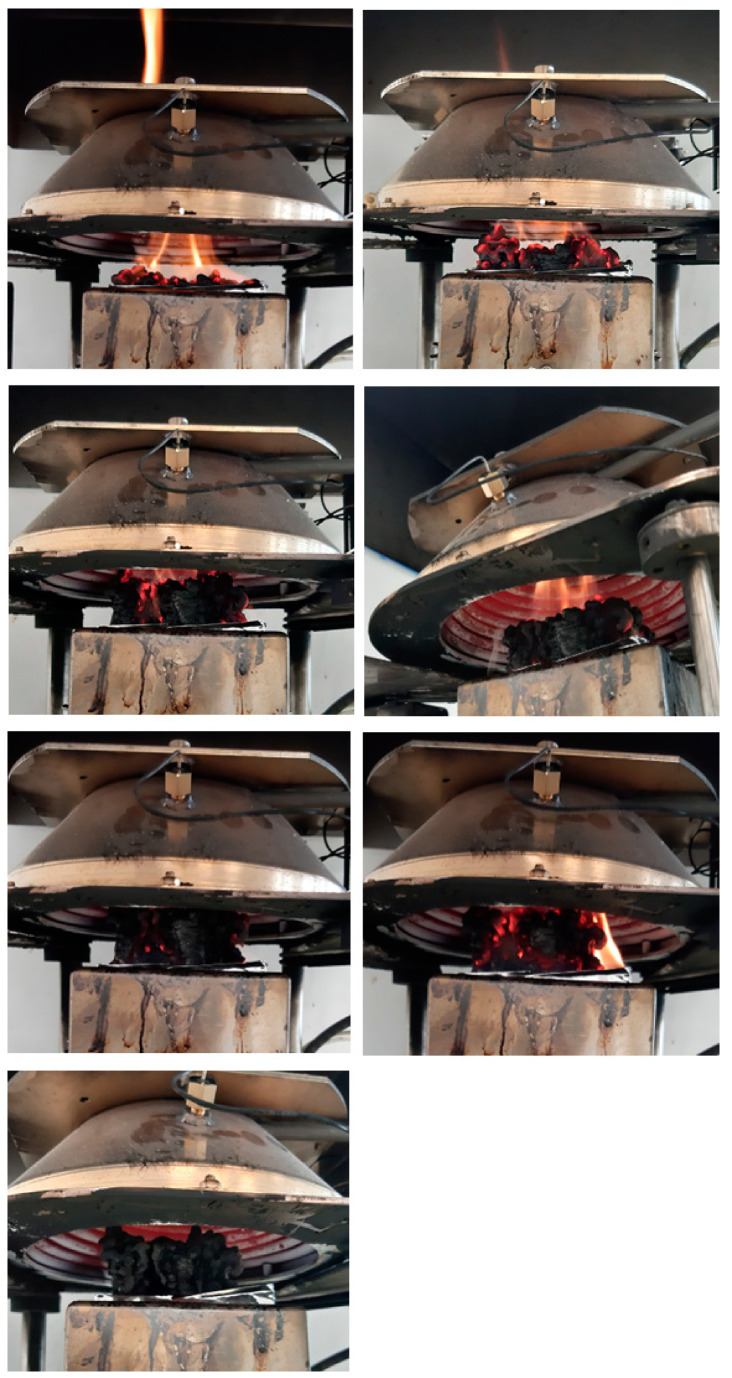
Burning behavior of TPS/10M during cone calorimetry tests.

Total smoke release (TSR) is a crucial parameter to evaluate the smoke emission during the cone calorimetry test. Figure 4B demonstrates that the replacement of part of the glycerol by urea and sorbitol caused a decrease in TSR. In the case of ternary plasticizer UGS, the maximum value of 46 m^2^/m^2^ was reached after several dozen seconds from the start of the test and remained at this level until the end of the measurement, while for G, the TSR was increasing, reaching a maximum of 261 m^2^/m^2^ in 470 s. Such reduction is mainly attributed to the capability of bonding unstable carbon particles in residue, as evidenced by the increased char yield, preventing them from escaping to the flame zone, as well as diluting by non-combustible gases. The photographic report made for TPS/10M during the cone calorimetry test, presented in Figure 5, demonstrates the creation of char on the surface of the polymer. A horizontally oriented sample subjected to the stream of heat flux underwent thermal decomposition and ignition. The employed modification resulted in forming a char layer, which was swelled and congealed, while growth char caused a reduction in the burning process intensity. At the end of the test, an increase in intensity, caused by a growth in the pyrolysis products emission due to a slight displacement of the sample, was observed. The photographs of the residue present a few centimetres high swollen structure and are consistent with the conducted analysis.

## 4. Conclusions

In the presented work mutual interactions between starch matrix, ternary deep eutectic solvent made with urea, glycerol and sorbitol (at molar ratio 2:1:1) used as plasticizer and two types of fillers: microcrystalline cellulose (as organic filler) and sodium montmorillonite (as inorganic filler) were investigated. DES effectively plasticized the polysaccharide leading to its highly amorphous structure. The introduction of the fillers improved mechanical properties, and decreased sorption degrees as well as WVTR values. As DMTA results indicated, the addition of the fillers did not affect the processability of the composites. XRD analysis revealed that the d-spacing of MMT-Na platelets was slightly enlarged in the polymer matrix after extrusion. SEM analysis confirmed that fillers were well embedded in TPS matrix. The cone calorimetry analysis of the TPS materials revealed that composite material plasticized with DES with the clay presence showed better fire retardant properties than TPS plasticized with a conventional plasticizer (glycerol).

## Figures and Tables

**Table 1:** Mechanical test results.

Sample	Tensile Strength [MPa]	Elongation at Break [%]	Young’s Modulus [MPa]	Thickness [mm]
TPS	4.6 (±0.26) ^d^	148 (±11.6) ^a^	112 (±14.3) ^d^	0.61 (±0.04) ^a^
TPS/5M	4.7 (±0.23) ^d^	122 (±7.2) ^b^	114 (±12.4) ^d^	0.55 (±0.00) ^b^
TPS/10M	6.2 (±0.30) ^a^	99 (±14.0) ^d^	232 (±15.2) ^a^	0.52 (±0.04) ^d^
TPS/5C	5.5 (±0.33) ^c^	124 (±10.0) ^b^	192 (±9.9) ^c^	0.57 (±0.04) ^c^
TPS/10C	5.9 (±0.32) ^b^	116 (±6.8) ^c^	211 (±16.4) ^b^	0.61 (±0.04) ^a^

a–d—averages marked with the same letters do not differ significantly from each other for *p* < 0.05.

**Table 2:** Swelling and dissolution degrees of TPS films distilled water, moisture adsorption degree and WVTR at 75% for 24 h.

Sample	Swelling Degree [%]	Dissolution Degree [%]	Moisture Sorption Degree at RH 75% [%]	WVTR_RH75%_ [g/m^2^·24 h]
TPS	87.4 (±0.9)	22.5 (±0.07)	24.0 (±0.07)	381 (±21.0)
TPS/M5	84.1 (±0.8)	21.6 (±1.02)	24.0 (±0.04)	352 (±8.2)
TPS/M10	71.1 (±0.0)	19.9 (±1.21)	23.6 (±0.40)	345 (±0.3)
TPS/C5	79.2 (±1.1)	20.9 (±1.15)	23.5 (±0.14)	369 (±3.2)
TPS/C10	77.7 (±9.9)	20.4 (±0.07)	23.0 (±0.08)	358 (±4.2)

**Table 3:** The cone calorimeter results of TPS/10M and TPS-G.

Sample	TTI [s]	PHRR [kW/m^2^]	MARHE [kW/m^2^]	PHRR/ t _PHRR_, [kW/m^2^s^1^]	THR [MJ/m^2^]	EHC [MJ/kg]	MLR [g/(s·m^2^)]
TPS/10M	79	132	71	1.6	44	12	5.3
TPS-G	54	317	167	4.9	60	15	9.2

## Data Availability

The data presented in this study are available on request from the corresponding author.
